# Does the Spectrum model accurately predict trends in adult mortality? Evaluation of model estimates using empirical data from a rural HIV community cohort study in north-western Tanzania

**DOI:** 10.3402/gha.v7.21783

**Published:** 2014-01-16

**Authors:** Denna Michael, Chifundo Kanjala, Clara Calvert, Carel Pretorius, Alison Wringe, Jim Todd, Balthazar Mtenga, Raphael Isingo, Basia Zaba, Mark Urassa

**Affiliations:** 1 Sexual and Reproductive Health Program, National Institute for Medical Research-Mwanza Center, Mwanza, Tanzania; 2 Department of Population Health, London School of Hygiene and Tropical Medicine (LSHTM), London, UK; 3 Futures Institute, Glastonbury, CT, USA

**Keywords:** HIV, adult mortality, Spectrum model, cohort

## Abstract

**Introduction:**

Spectrum epidemiological models are used by UNAIDS to provide global, regional and national HIV estimates and projections, which are then used for evidence-based health planning for HIV services. However, there are no validations of the Spectrum model against empirical serological and mortality data from populations in sub-Saharan Africa.

**Methods:**

Serologic, demographic and verbal autopsy data have been regularly collected among over 30,000 residents in north-western Tanzania since 1994. Five-year age-specific mortality rates (ASMRs) per 1,000 person years and the probability of dying between 15 and 60 years of age (_45_Q_15_,) were calculated and compared with the Spectrum model outputs. Mortality trends by HIV status are shown for periods before the introduction of antiretroviral therapy (1994–1999, 2000–2005) and the first 5 years afterwards (2005–2009).

**Results:**

Among 30–34 year olds of both sexes, observed ASMRs per 1,000 person years were 13.33 (95% CI: 10.75–16.52) in the period 1994–1999, 11.03 (95% CI: 8.84–13.77) in 2000–2004, and 6.22 (95% CI; 4.75–8.15) in 2005–2009. Among the same age group, the ASMRs estimated by the Spectrum model were 10.55, 11.13 and 8.15 for the periods 1994–1999, 2000–2004 and 2005–2009, respectively. The cohort data, for both sexes combined, showed that the _45_Q_15_ declined from 39% (95% CI: 27–55%) in 1994 to 22% (95% CI: 17–29%) in 2009, whereas the Spectrum model predicted a decline from 43% in 1994 to 37% in 2009.

**Conclusion:**

From 1994 to 2009, the observed decrease in ASMRs was steeper in younger age groups than that predicted by the Spectrum model, perhaps because the Spectrum model under-estimated the ASMRs in 30–34 year olds in 1994–99. However, the Spectrum model predicted a greater decrease in _45_Q_15_ mortality than observed in the cohort, although the reasons for this over-estimate are unclear.

Tanzania, like many other sub-Saharan African countries, has experienced high adult mortality due to HIV/AIDS. The adult mortality rate is the probability of dying between age 15 and 60 years and is known to demographers as _45_Q_15_. In Tanzania _45_Q_15_ was estimated as 45% in 1995, 45% in 2000, and 40% in 2005 (1), but in a study in northern Tanzania in 1999, the _45_Q_15_ was estimated as 49% for men and 46% for women (2). There is strong evidence that all-cause, adult mortality rates elsewhere in East Africa have declined over the past 10 years, with Uganda showing that all-cause adult mortality declined by 25% following the roll-out of anti-retroviral therapy (ART) (3). Other sites in Africa compare all-cause adult mortality 2–6 years following ART roll-out with pre-ART period and show a decrease of 42% in Karonga, Malawi; 21% in Kisesa, Tanzania; and 21% in UMkhanyakude, South Africa (4), although the overall pattern in South Africa may not show much decrease in all-cause adult mortality (5). However, HIV is still a leading cause of adult death in many countries in East and Southern Africa (6).

In Tanzania in 1996, in a population aged 15–54 years with a 4% prevalence of HIV, 35% of all deaths occurred in HIV positives (7). In Zambia in 1999 in two townships with a 15% prevalence of HIV among adults, 53% of all deaths were attributable to HIV (8) while in Zimbabwe, in 2006, 61% of all adult deaths were attributable to HIV (9). However, HIV-attributable mortality has declined in Tanzania since 2004 (C. Kanjala et al., unpublished data), and the most obvious reason for the decline in all-cause and HIV-attributable mortality is the availability of ART (10).

Due to the weaknesses in vital registration and health information systems in developing countries, little information is available for planning of health services. National and regional health planners depend on estimates and projections from demographic and epidemiological models. Even if there were reliable demographic data in these countries, good, validated models would be needed to study the potential impact of health interventions. The Spectrum model, developed by the Futures Institute, uses information from national or sentinels, population-based surveys, in order to estimate key parameters of the HIV epidemic (11, 12). Estimating HIV incidence from prevalence relies on an accurate progression and survival model for HIV, which has thus far been validated against a limited number of data sets. Problems with data quality and model specification can lead to incorrect projections. Demographic surveillance sites that also collect HIV data can be used to verify the model specification, and validate the out-parameters of the Spectrum model, thus improving the model fit, and the understanding of the HIV epidemic curves applied to the data.

This paper describes trends and patterns in age-specific mortality rates (ASMRs) and in the index of adult mortality (_45_Q_15_) in Kisesa HIV cohort study in north-western Tanzania from 1999 to 2009. The paper then compares these observed mortality rates and _45_Q_15_ with estimates from the Spectrum model.

## Methods

### Population data

The Kisesa HIV cohort study is based on the population of seven villages located approximately 20 km east of Mwanza City in north-western Tanzania. The seven villages range from urbanised, roadside settlements near the ward's trading centre to remote rural settlements, with 95% of the population belonging to the Sukuma tribe, the largest ethnic group in Tanzania. The main economic activities are small-scale farming and petty trade of both agricultural and livestock products, while a minority of residents are fishermen. The demographic surveillance system (DSS) conducts a household count every 3–6 months to record new residents through births and in-migration, as well as those lost to the cohort through death and out-migration. The DSS began in 1994, with a population of 19,350, and by 2011 the population had increased to 34,000 (13). Over the same period (1994–2011), six serological surveys have been conducted, 2–3 years apart, to monitor trends in the HIV epidemic in adults aged 15 years and over in the population (14, 15). Dry blood spots are collected and two independent enzyme-linked immunosorbent assays are used, according to Tanzanian guidelines to determine those who are HIV positive.

### Data management and statistical analysis

All data from community cohort were entered using Census and Surveys Processing (CSPro) software (U.S. Bureau of the Census & ORC Macro's MEASURE *DHS*+project, 2000), and transferred to STATA Version12 (Stata Corp., College Station, Texas) for analysis. ASMRs were calculated by dividing the number of deaths observed by the person years of follow-up, for each sex and 5-year age group in the resident population. Adult mortality was summarized as _45_Q_15_, which is obtained by multiplying the survival derived from the ASMRs. The _45_Q_15_ measure was obtained separately for males and females in the cohort, and based on the serological HIV status of those who had been tested, the _45_Q_15_ measure was obtained for HIV-positive, HIV-negative and HIV-unknown groups. Parameters, ASME and _45_Q_15_, estimated from the cohort data are shown with 95% confidence intervals (95% CI) derived from the Poisson distribution for deaths in this cohort.

### The Spectrum model

The Spectrum model analyses existing country-specific data to estimate the current and future population structure and health of the people in the country. It comprises of different, integrated policy modules such as the Demographical Projection (DemProj), AIDS Impact (AIM), Family Planning (FamPlan), Child Survival (LIST) among others. Each module relies on programmatic input data and demographical information obtained from DemProj (which in turns bases its projection on data from the UN Population Division) to produce indicators for a given public health area.

AIM is at the root of the Spectrum estimates for HIV-attributable mortality. It consists of three independent sub-models: mother-to-child transmission, child HIV and adult HIV. AIM projects the consequences of HIV incidence trends, including the number of people living with HIV, number of new infections, number of pregnant women infected with HIV, and mortality due to AIDS. It is widely used to forecast near-term treatment and prevention of mother-to-child transmission (PMTCT) needs. In addition to the role AIM plays in HIV program planning, it is also used to estimate the impact of HIV prevention programs, such as the impact of expanding PMTCT and ART programs.

The Spectrum model used the general patterns of the HIV epidemic in Tanzania, with changes to specifically model the age and sex structure in Kisesa. The specific input parameters to the Spectrum model were obtained from a workshop in 2012 with members of the network for Analysis of Longitudinal Population-based HIV data in Africa (ALPHA). Details of data analysis work by the ALPHA network are given by Maher et al. (16). The structure of the Spectrum modelling and the general parameters are described elsewhere (17).

### Ethical issues

Individual informed consent was obtained from sero survey study participants. Research activities in the Kisesa cohort study were granted ethics approval by the Tanzanian National Research Ethics Review Sub-committee (NatREC) and the London School of Hygiene and Tropical Medicine (LSHTM) ethics committee.

## Results

### Adult ASMRs

**Fig. 1:**
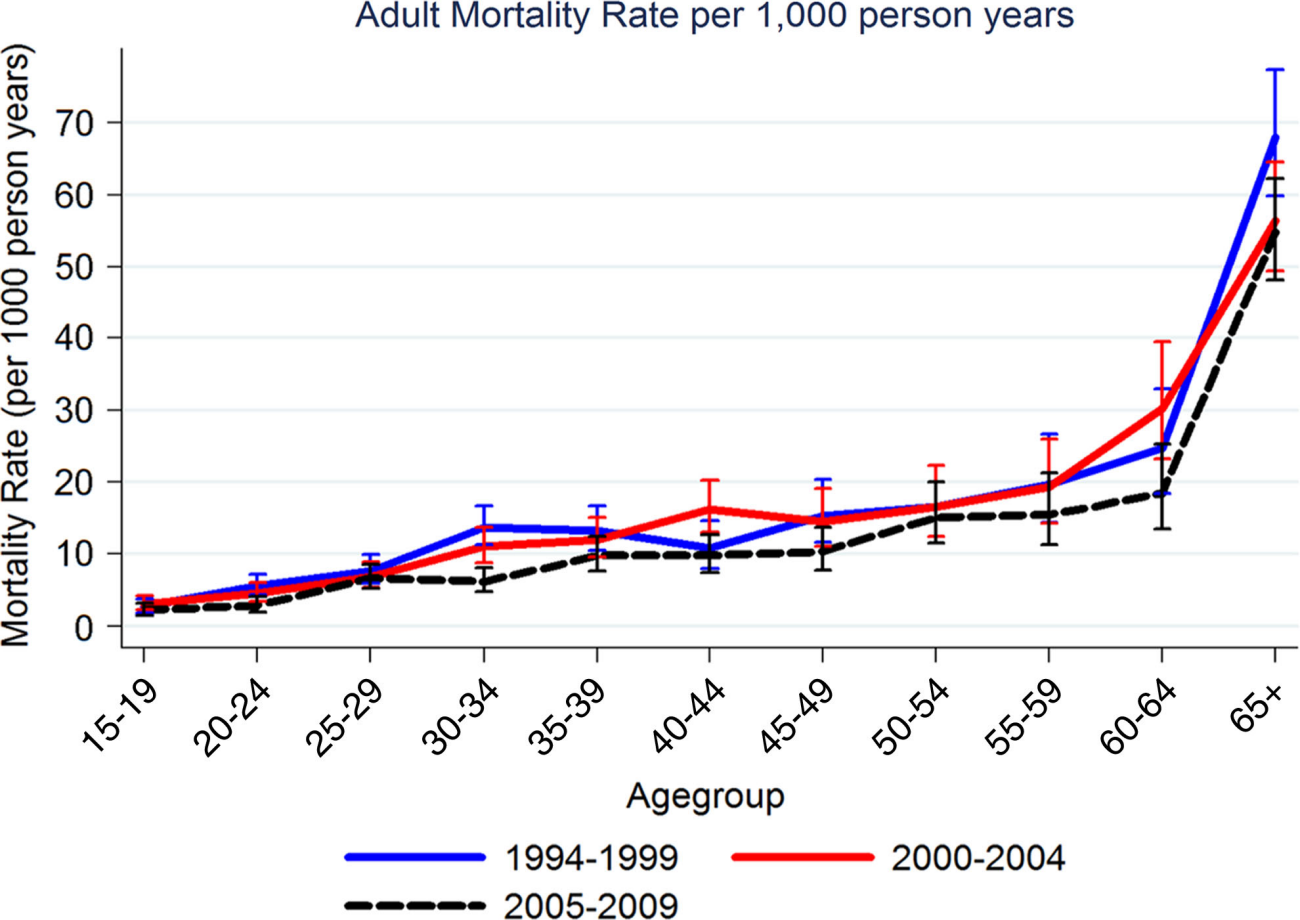
Adult age-specific mortality rates per 1,000 person years (all-cause, both sexes) estimated in Kisesa DSS, 1994–2009.

Figure 1 shows the ASMRs for three periods (1994–1999, 2000–2004, 2005–2009). ASMRs per 1,000 person years among those aged 30–34 were 13.33 (95% CI: 10.75–16.52) in the period 1994–1999, 11.03 (95% CI: 8.84–13.77) in 2000–2004, and 6.22 (95% CI: 4.75–8.15) in 2005–2009. Overall all-cause mortality rates in Kisesa are lower in the period 2005–2009 than in previous periods for all age groups, although the 95% confidence intervals are wide and the differences for any age group may not be significant. In all periods, all-cause mortality increased with age, with ASMRs for three periods (1994–1999, 2000–2004 and 2005–2009) shown separately for men and women (Table 1). The Spectrum model showed that ASMRs for adults aged 30–49 years increased between the 1994–1999 period and the 2000–2004 period. For all ages the ASMRs decreased between 2000–2004 and 2005–2009.

**Table 1:** Adult age-specific mortality rates per 1,000 person years (all-cause, men and women) from Kisesa cohort data and Spectrum model estimates

	1994–1999				2000–2004				2005–2009			
			
	Empirical data			Spectrum estimate	Empirical data			Spectrum estimate	Empirical data			Spectrum estimate
						
Age group	Number of deaths	Number of person years	Mortality rate (95% CI)	Mortality rate	Number of deaths	Number of person years	Mortality rate (95% CI)	Mortality rate	Number of deaths	Number of person years	Mortality rate (95% CI)	Mortality rate
**Men**												
15–19	19	6,064	3.13 (2–4.91)	3.06	19	6,275	3.03 (1.93–4.75)	2.69	15	7,654	1.96 (1.18–3.25)	2.31
20–24	19	4,716	4.03 (2.57–6.32)	4.86	17	5,029	3.38 (2.1–5.44)	4.23	20	5,229	3.82 (2.47–5.93)	3.58
25–29	25	3,615	6.91 (4.67–10.23)	7.51	24	4,089	5.87 (3.93–8.76)	6.81	31	4,165	7.44 (5.23–10.58)	5.60
30–34	49	3,312	14.80 (11.18–19.58)	10.24	32	3,344	9.57 (6.77–13.53)	10.09	28	4,096	6.84 (4.72–9.90)	8.07
35–39	45	2,654	16.96 (12.66–22.71)	13.95	33	2,922	11.30 (8.03–15.89)	14.60	37	3,249	11.39 (8.25–15.72)	11.85
40–44	21	1,903	11.04 (7.2–16.93)	15.02	49	2,425	20.21 (15.27–6.74)	16.14	31	2,744	11.30 (7.94–16.06)	13.10
45–49	31	1,609	19.26 (13.55–27.39)	15.38	31	1,688	18.36 (12.91–26.11)	16.09	25	2,254	11.09 (7.49–16.41)	13.55
50–54	25	1,333	18.76 (12.68–27.76)	15.29	25	1,290	19.39 (13.1–28.69)	14.90	31	1,563	19.83 (13.94–28.19)	12.73
55–59	23	1,005	22.89 (15.21–34.44)	25.15	24	1,093	21.95 (14.71–32.75)	23.61	21	1,147	18.31 (11.94–28.08)	19.97
60 +	126	2,387	52.79 (40.33–69.79)	62.28	138	2,500	55.20 (42.35–71.80)	59.95	135	2,801	48.20 (36.34–63.88)	56.86
**Women**												
15–19	10	5,265	1.90 (1.02–3.53)	2.59	19	5,774	3.29 (2.1–5.16)	2.23	17	6,881	2.47 (1.54–3.97)	1.80
20–24	35	4,983	7.02 (5.04–9.78)	4.81	28	4,954	5.65 (3.9–8.19)	4.23	10	5,310	1.88 (1.01–3.5)	3.43
25–29	35	4,164	8.40 (6.03–11.71)	8.97	36	4,572	7.87 (5.68–10.92)	8.71	29	4,767	6.08 (4.23–8.75)	6.82
30–34	46	3,598	12.78 (9.58–17.07)	10.85	46	3,728	12.34 (9.24–16.47)	12.17	25	4,423	5.65 (3.82–8.37)	9.44
35–39	25	2,619	9.55 (6.45–14.13)	12.15	40	3,164	12.64 (9.27–17.24)	14.16	28	3,402	8.23 (5.68–11.92)	11.87
40–44	22	2,059	10.69 (7.04–16.23)	11.48	30	2,436	12.31 (8.61–17.61)	13.04	25	2,995	8.35 (5.64–12.35)	10.94
45–49	18	1,566	11.50 (7.24–18.25)	14.00	21	1,884	11.15 (7.27–17.1)	14.80	23	2,379	9.67 (6.42–14.55)	12.35
50–54	19	1,368	13.89 (8.86–21.78)	14.30	20	1,406	14.22 (9.18–22.04)	13.74	19	1,733	10.97 (6.99–17.19)	11.30
55–59	19	1,079	17.62 (11.24–27.62)	13.84	20	1,186	16.87 (10.88–26.14)	12.63	18	1,363	13.21 (8.32–20.97)	10.71
60+	151	2,866	52.69 (40.21–69.00)	55.60	132	3,113	42.40 (36.31–48.81)	53.60	139	3,612	38.48 (33.45–4497)	51.03

### _45_Q_15_ mortality trends

Table 2 shows time trends of the index of the proportion of adult deaths between 15 and 60 years of age (_45_Q_15_) by sex from the Kisesa DSS data, and the Spectrum model estimates. The probability of dying in the age group 15–60 has declined from the year 2000 for both sexes. The chance of dying in the 15–60 age group for both sexes combined was 39% (95% CI: 27–55%) in 1994 and 22% (95% CI: 15–31%) in 2009. Men were found to have a higher _45_Q_15_ than women across all time periods, though the 95% confidence intervals overlap in each of the years. With ART introduction in 2005, there is a consistent decline in the _45_Q_15_ measure over the next 5 years.

**Table 2:** Index of adult mortality (_45_Q_15_) by sex and calendar year from Kisesa cohort data and Spectrum model estimates

	Men			Women			Both sexes		
			
Year	Empirical _45_Q_15_ α (95% CI)	Spectrum estimates	Differenceγ	Empirical _45_Q_15_ α (95% CI)β	Spectrum estimates	Differenceγ	Empirical _45_Q_15_ α (95% CI)	Spectrum estimates	Differenceγ
1994	0.43 (0.26–0.63)	0.47	0.04	0.35 (0.18–0.61)	0.40	0.05	0.39 (0.27–0.55)	0.43	0.04
1995	0.30 (0.21–0.40)	0.48	0.18	0.43 (0.32–0.55)	0.41	−0.02	0.36 (0.29–0.45)	0.45	0.09
1996	0.42 (0.32–0.53)	0.49	0.07	0.42 (0.31–0.54)	0.43	0.01	0.41 (0.34–0.49)	0.46	0.05
1997	0.53 (0.42–0.64)	0.50	−0.03	0.36 (0.27–0.48)	0.44	0.08	0.45 (0.38–0.54)	0.47	0.02
1998	0.37 (0.27–0.50)	0.50	0.13	0.34 (0.23–0.48)	0.45	0.11	0.35 (0.28–0.44)	0.48	0.13
1999	0.58 (0.46–0.70)	0.50	−0.08	0.34 (0.26–0.45)	0.46	0.12	0.47 (0.39–0.55)	0.48	0.01
2000	0.46 (0.35–0.57)	0.50	0.04	0.42 (0.34–0.51)	0.46	0.04	0.44 (0.37–0.51)	0.48	0.04
2001	0.42 (0.32–0.53)	0.49	0.07	0.39 (0.30–0.50)	0.46	0.07	0.41 (0.34–0.49)	0.48	0.07
2002	0.33 (0.24–0.44)	0.49	0.16	0.35 (0.27–0.45)	0.46	0.11	0.34 (0.28–0.41)	0.47	0.13
2003	0.45 (0.35–0.56)	0.48	0.03	0.35 (0.27–0.46)	0.46	0.11	0.40 (0.33–0.48)	0.47	0.07
2004	0.43 (0.34–0.54)	0.47	0.04	0.32 (0.24–0.43)	0.45	0.13	0.38 (0.32–0.45)	0.46	0.08
2005	0.40 (0.31–0.50)	0.46	0.06	0.36 (0.28–0.46)	0.44	0.12	0.38 (0.32–0.45)	0.45	0.07
2006	0.35 (0.26–0.47)	0.45	0.10	0.22 (0.14–0.32)	0.43	0.11	0.29 (0.22–0.36)	0.44	0.15
2007	0.46 (0.35–0.59)	0.43	−0.03	0.31 (0.22–0.41)	0.40	0.09	0.38 (0.31–0.46)	0.41	0.03
2008	0.30 (0.21–0.41)	0.40	0.10	0.25 (0.16–0.36)	0.37	0.12	0.27 (0.20–0.34)	0.38	0.11
2009	0.26 (0.18–0.38)	0.38	0.12	0.18 (0.13–0.26)	0.35	0.17	0.22 (0.17–0.29)	0.37	0.15

α 45q15 denotes probability of dying between 15–60 years of age.

β CI denotes the 95% confidence intervals.

γ Difference=Spectrum _45_Q_15_ minus Empirical _45_Q_15_.

The Spectrum model showed an increase in the _45_Q_15_ measure of mortality for both sexes from 43% in 1994 to 48% in 2000 and a subsequent decline to 37% in 2009. A similar pattern was shown for women and men separately, with men having a higher probability of dying than women across all periods of time. Figure 1 illustrates trends in the index of adult mortality (_45_Q_15_) by sex. The _45_Q_15_ was generally higher in men compared to women except in a few years. The difference in _45_Q_15_ measure of mortality between empirical data and the modelled estimate ranged from 0.03 to 0.16 among men and 0.01 to 0.17 among women. Figure 3 illustrates how the two data sources, which are empirical versus modelled data for index of adult mortality (_45_Q_15_) by sex and calendar year, compare to each other.

### Adult mortality (_45_Q_15_) trends by HIV status

**Fig. 2:**
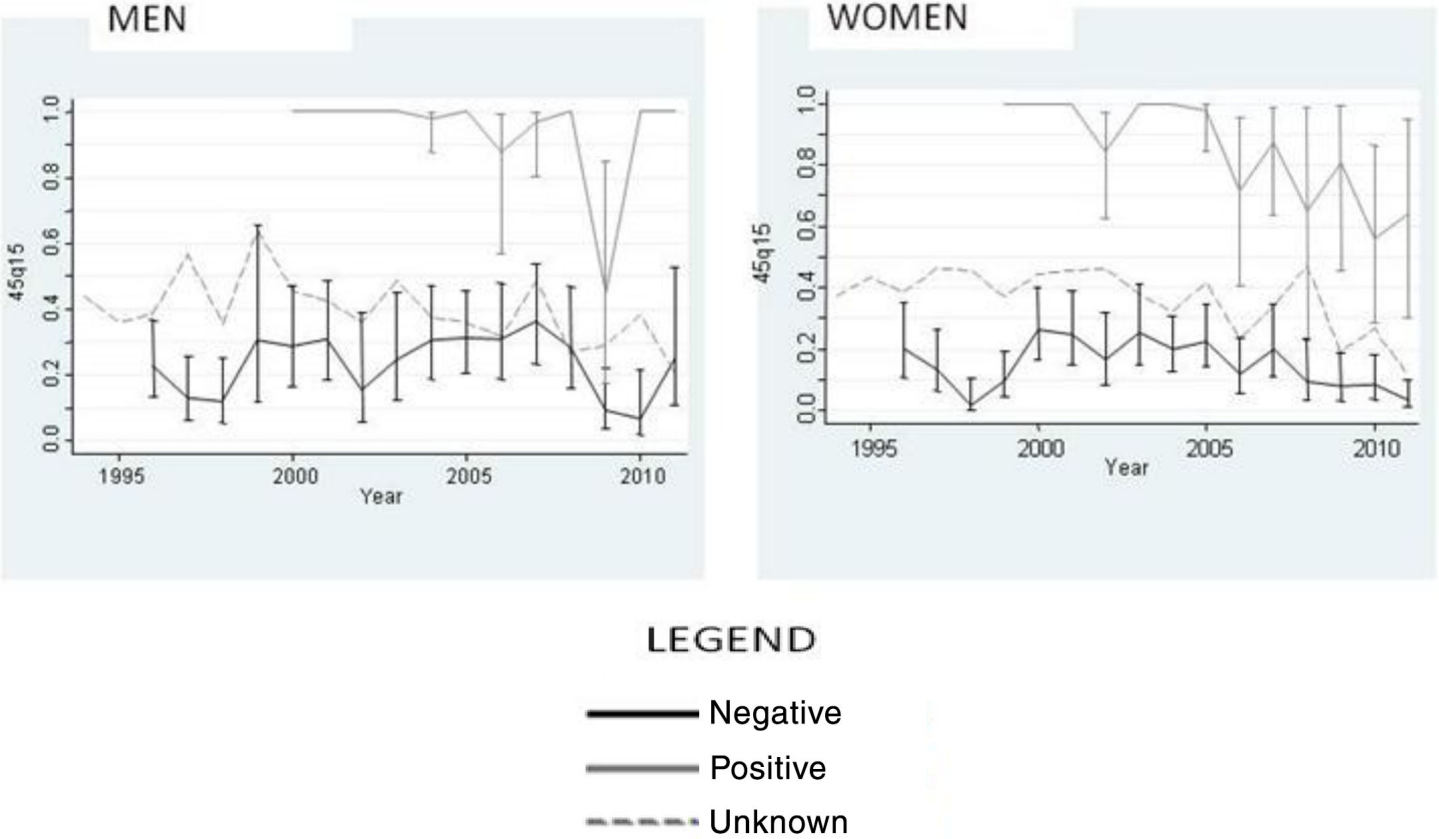
Index of adult mortality by HIV status and calendar year with 95% confidence intervals.

Figure 2 illustrates trends in _45_Q_15_ by HIV status separately for men and women. The highest _45_Q_15_ for men was reported in 1999 (58%, 95%CI: 46–70%) whilst the highest _45_Q_15_ for women was in 2000 (42%, 95%CI: 34–51%). _45_Q_15_ for HIV-positive adults was consistently higher than HIV-negative adults across all time periods. Adults with unknown sero status had higher _45_Q_15_ than HIV-negatives but lower than HIV-positive adults. The trend was the same for both men and women. Although not conclusive, there was a downward trend of _45_Q_15_ when both sexes were combined regardless of the sero status. This downtrend was more pronounced among the HIV-positive females than among the HIV-positive males.

**Fig. 3:**
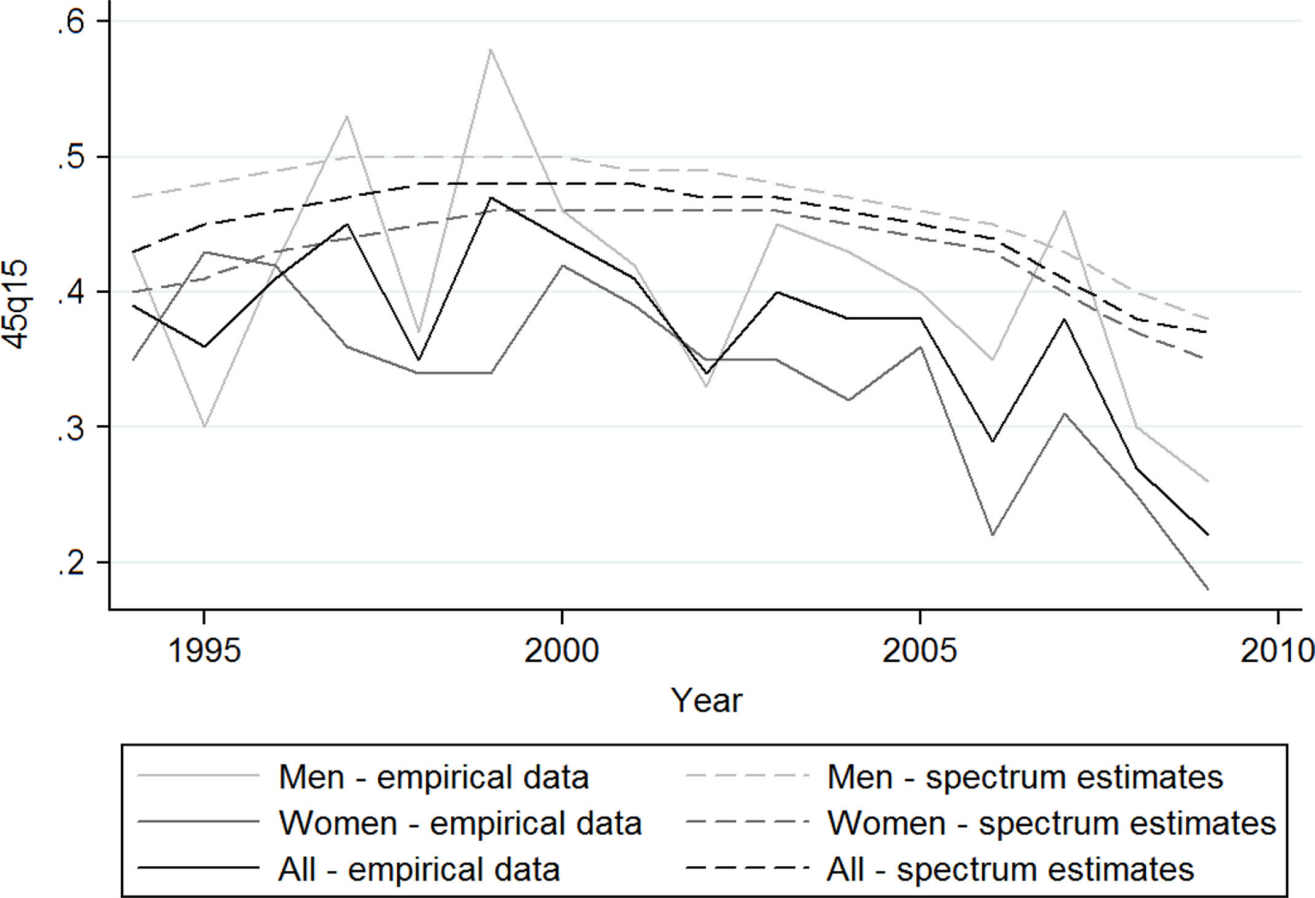
Index of adult mortality (_45_Q_15_) by sex and calendar year from Kisesa cohort data and Spectrum model estimates.

## Discussion

Adult ASMRs in Kisesa peaked during the mid-1990s and declined during the periods from 2000 onwards. As expected ASMRs increased with increasing age, and the observed pattern of ASMRs corresponds to the expected mortality patterns in HIV infected adults (18). ART became available in Tanzania in 2005 through the care and treatment clinics (CTC) and the first members of the Kisesa cohort were referred to CTC in 2005. Uptake of ART in this cohort among those estimated to be in need of treatment has been estimated at less than 3% (19), but substantial declines in ASMRs have been seen, especially among adults aged 30–49 years. This is similar to the declines in ASMRs seen in other countries in sub-Saharan Africa following the introduction of ART (3).

The probability of dying between 15 and 60 years of age (_45_Q_15_) also showed a peak in the mid-1990s with a subsequent decrease of around 25% through to 2009. The agreement of the empirical data with the Spectrum model estimates for _45_Q_15_ show the expected epidemic curve for HIV peaking in the mid-1990s, and the larger declines from 2005 due to the introduction of ART in the community. This analysis shows good agreement between the outputs from the Spectrum model and the ASMRs and _45_Q_15_ estimated from the empirical data using HIV serology. The model-based results for these measures of adult mortality in some population segments are smoothed, and the empirical results show variation from year to year, due to the relatively small numbers of deaths observed in this rural community cohort. Further work is needed to evaluate the model with community-based mortality data from other serological and verbal autopsy studies.

For the estimates of _45_Q_15_ in this rural cohort, the agreement between the serological estimates and those obtained from verbal autopsy using the Inter-VA4 software were less convincing. Whereas the serological data showed a marked drop in the proportion dying in 2005, after the introduction of ART, the verbal autopsy results showed little change after 2005 (C. Kanjala et al., unpublished data). In this supplement, Oti validates the Spectrum model against verbal autopsy data interpreted through Inter-VA4 in Nairobi and concludes that both HIV and TB deaths from the model need to be included to give an agreement with the Spectrum results (20). This may also be the reason for poor correlation between the empirical and Spectrum-modelled results in Kisesa.

In 2004, Stover et al. validated the Spectrum model estimates against empirical estimates of mortality from six population-based studies and concluded that the model performed well (21). However, there are few reports of validation of the Spectrum model against empirical serological data from populations in sub-Saharan Africa. A previous report noted that model-based approaches may over-estimate mortality due to HIV and may therefore over-estimate the decline in mortality with the introduction of ART (22).

In this analysis, estimated ASMRs in adults from the empirical data agreed well with the estimates from the Spectrum model. In particular, the decline in mortality seen in adults aged 30–49 years from 2005 onwards provides some evidence of the impact of ART in this population. A pooled analysis of population-based data in four selected sites in East and Southern Africa depicted a similar trend in adult mortality, which is greatly influenced by the introduction of ART in these areas (3, 19, 23). These results suggest that mortality trends and patterns may have been adequately modelled and predicted by the Spectrum model.

The observed data on the probability of dying between 15 and 60 years of age in this analysis were similar to the estimates from a population-based study in Uganda. In Uganda, the _45_Q_15_ before 2004 was estimated as 51% for men (95% CI: 45–57%) and 44% for women (95% CI: 39–49%), decreasing from 2005 onwards, to 38% (95% CI: 33–44%) for men and 32% (95% CI: 28–37%) for women (3). Although there was a slightly higher overall estimate of _45_Q_15_ in Uganda compared to these results, there is a consistent trend in a decreasing _45_Q_15_ following the introduction of ART.

A comparison of the _45_Q_15_ estimates for those known to be HIV positive reinforces the conclusion that mortality has decreased, and that a greater decrease has been observed in women than men. From 2005, empirical data show that the _45_Q_15_ in women has been declining, indicating that survival in those that are HIV positive has improved. It is not surprising that women have shown greater improvements in survival, as it has been shown that women are more likely to access ART relative to estimated need for treatment and that they access ART with higher CD4 counts and better immunological status (24).

The data for this paper have been taken from demographic surveillance of vital events in 30,000 people since 1994. With less than 100 deaths per annum between the ages of 15 and 60 years, there is considerable variation in the annual adult mortality. Previous studies have shown that epidemiological models may over-estimate mortality due to HIV/AIDS, although some deaths may have been missed from this study due to out-migration. We have presented the data on ASMRs and _45_Q_15_ in this paper, and have not shown age adjusted mortality rates, as the population structure in Kisesa has not changed much over the 19 years of follow-up, and the age adjusted adult mortality rate is very similar to the _45_Q_15_ that we present. Although the size of the decline in all-cause mortality may differ between the model and the observed data, the decline from pre-ART to post-ART periods is clear from both the data and the model outputs.

## Conclusion

Most resource-limited countries lack vital registration data, in order to estimate mortality rates and other demographic population parameters. This article presents a fair comparison of mortality estimates generated by the Spectrum model against empirical data from a long-standing population-based cohort. From 1994 to 2009, the observed decrease in ASMRs was steeper in younger age groups than that predicted by the Spectrum model perhaps because the Spectrum model under-estimated the ASMRs in 30–34 year olds in 1994–99. However, the Spectrum model predicted a greater decrease in _45_Q_15_ mortality than observed in the cohort, although the reasons for this over-estimate are unclear. These findings have evidently shown a technical gap to be filled to improve the Spectrum model such that it can exactly predict population parameters in setting where empirical data are scarce but they are needed for health planning.
