# Corrigendum

**DOI:** 10.3402/gha.v7.24079

**Published:** 2014-03-11

Regarding the paper titled: ‘Does the Spectrum model accurately predict trends in adult mortality? Evaluation of model estimates using empirical data from a rural HIV community cohort study in north-western Tanzania’ by *Denna Michael, Chifundo Kanjala, Clara Calvert, Carel Pretorius, Alison Wringe, Jim Todd, Balthazar Mtenga, Raphael Isingo, Basia Zaba, Mark Urassa*

Published in *Global Health Action* (section “HIV Mortality”) 16 Jan 2014. Citation: Glob Health Action 2014, **7**: 21783 - http://dx.doi.org/10.3402/gha.v7.21783

The last sentences of the Abstract is incorrect:

It currently reads: “However, the Spectrum model predicted **a greater decline in** 45Q15 mortality than observed in the cohort, although the reasons for this over-estimate are unclear.”

This sentence should read: “*However, the Spectrum model predicted **greater** 45Q15 mortality than observed in the cohort, although the reasons for this over-estimate are unclear*.”

The complete abstract is displayed below:

***Introduction***: Spectrum epidemiological models are used by UNAIDS to provide global, regional and national HIV estimates and projections, which are then used for evidence-based health planning for HIV services. However, there are no validations of the Spectrum model against empirical serological and mortality data from populations in sub-Saharan Africa.

***Methods***: Serologic, demographic and verbal autopsy data have been regularly collected among over 30,000 residents in north-western Tanzania since 1994. Five-year age-specific mortality rates (ASMRs) per 1,000 person years and the probability of dying between 15 and 60 years of age (_45_Q_15_,) were calculated and compared with the Spectrum model outputs. Mortality trends by HIV status are shown for periods before the introduction of antiretroviral therapy (1994–1999, 2000–2005) and the first 5 years afterwards (2005–2009).

***Results***: Among 30–34 year olds of both sexes, observed ASMRs per 1,000 person years were 13.33 (95% CI: 10.75–16.52) in the period 1994–1999, 11.03 (95% CI: 8.84–13.77) in 2000–2004, and 6.22 (95% CI; 4.75–8.15) in 2005–2009. Among the same age group, the ASMRs estimated by the Spectrum model were 10.55, 11.13 and 8.15 for the periods 1994–1999, 2000–2004 and 2005–2009, respectively. The cohort data, for both sexes combined, showed that the _45_Q_15_ declined from 39% (95% CI: 27–55%) in 1994 to 22% (95% CI: 17–29%) in 2009, whereas the Spectrum model predicted a decline from 43% in 1994 to 37% in 2009.

***Conclusion***: From 1994 to 2009, the observed decrease in ASMRs was steeper in younger age groups than that predicted by the Spectrum model, perhaps because the Spectrum model under-estimated the ASMRs in 30–34 year olds in 1994–99. However, the Spectrum model predicted greater 45Q15 mortality than observed in the cohort, although the reasons for this over-estimate are unclear.

